# A modified rat model of isolated bilateral pulmonary contusion

**DOI:** 10.3892/etm.2012.615

**Published:** 2012-06-18

**Authors:** SHAOHUA WANG, ZHENG RUAN, JIE ZHANG, JIN ZHENG

**Affiliations:** 1 Departments of Thoracic Surgery,; 2 Radiology and; 3 Pathology, The First People’s Hospital Affiliated to Shanghai Jiao Tong University, Shanghai 200080, P.R. China

**Keywords:** lung, computed tomography, blunt chest trauma, pulmonary contusion

## Abstract

The aim of the present study was to create a feasible specific rat model of isolated bilateral pulmonary contusion (PC) and to evaluate the relationship between severity of hypoxemia and quantity of contusion lesions. Anesthetized rats were placed in a prone position. Injury energy ranging from 2.1 to 3.0 J was produced by a falling weight passed through a specially designed arched shield to the bilateral chest wall of rats. After injury (4 h), the contusion volume was measured using computer-generated three-dimensional reconstruction from a chest computed tomographic scan and expressed as a percentage of total lung volume. Arterial partial pressure of oxygen (PaO_2_) in blood gas analysis and contusion volume percentage were used to assess the severity of contusion. Heart and lung biopsy was used to confirm the diagnosis and rule out the existence of myocardial contusion. There were 3 cases of death and 1 case of death in the 3.0 J and the 2.4 J group, respectively. PaO_2_ in the 2.7 J group was significantly lower than that in the lower energy groups (P<0.001). The percentage of pulmonary contusion in the 2.7 J group was significantly higher compared to that of the lower energy groups (P<0.001). PaO_2_ was negatively correlated with contusion percentage (R^2^=0.76). Hemorrhage, edema and neutrophil infiltration were determined by lung biopsy. No evidence of myocardial contusion was documented in multiple heart biopsies. The method illustrated in this research effectively duplicates isolated bilateral pulmonary contusion in rats, the severity of which is highly correlated with the contusion size. Thus, 2.7 J can be regarded as the maximal energy for sublethal injury.

## Introduction

Blunt chest trauma is involved in approximately 1 of 3 acute trauma admissions to the hospital, and pulmonary contusion (PC) is an independent risk factor for the development of acute lung injury (ALI), acute respiratory distress syndrome (ARDS) and ventilator-associated pneumonia (VAP). Moreover, PC is the major cause of mortality after blunt chest trauma (1). Clinically, it is common that patients with approximately the same pulmonary contusion area have different manifestations, which may be relevant to the complex condition of the injured patients. Inflammatory molecular mechanisms, which are unable to be demonstrated by imaging tools, may be involved in the pathophysiology of PC. Research on the pathophysiology of PC is helpful for early diagnosis and therapy, reducing mortality and morbidity. Various models for PC reported in the literature utilize large animals such as canines, swine and monkeys. Although more analogous to the human, these models are costly. Even worse, the lack of molecular probes and other cell- and mediator-specific reagents, which are much more widely available for small animals such as mice and rats, limits the widespread use of large animal models in the study of PC pathophysiology. Most rodent models for PC in the literature are unilateral PC models and are complicated by the coexistence of myocardial contusion, which is likely to have an effect on the interpretation of the experimental results. The bilateral PC model designed by Raghavendran *et al* (2) is less likely to cause myocardial contusion, however, the experimental platform is complex and relatively costly. The correlation between pathophysiological changes and contusion lesions in images in rat models has not been well established. In this study, we aimed to develop a modified feasible rat model for bilateral contusion and to determine the most suitable injury energy in order to further reduce the possibility of myocardial contusion, which will provide a simple and reliable animal model for the further study of PC.

## Materials and methods

### Experimental animals

Adult Sprague-Dawley rats (n=36; 300–350 g; male) (Shanghai SLAC Laboratory Animal, Co., Ltd.) were equally divided into 4 groups. PC was induced with different injury energies in these 36 rats. Another 9 rats comprised the comparative group.

### Principles of energy production and control

**Figure 1:**
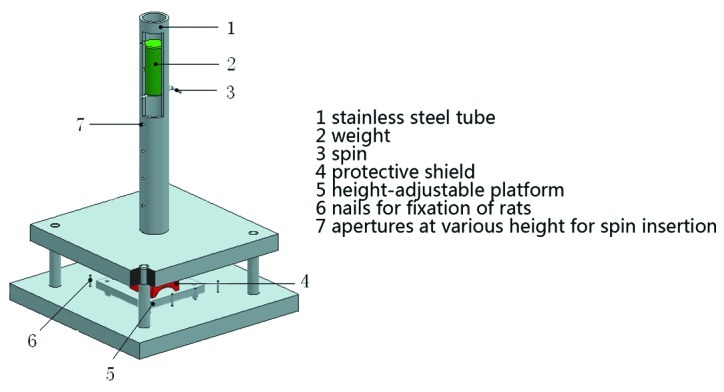
Schematic diagram of the experimental device.

The rat model for bilateral PC was induced by high-fall energy. The experimental device is illustrated in Fig. 1. A hollow aluminum cylindrical weight was dropped freely from various heights (controlled by a spin) through a vertical lubricated stainless steel tube onto a protective shield resting on the dorsum of the rats, which was fixed on a height-adjustable steel platform. The injury energy was calculated using the following equation: E = mgh, where m is the mass of the aluminum weight (kg), g is the gravitational acceleration (10 m/s^2^ to facilitate calculation) and h is the height of spin above the protective shield (m). A key feature of the model was the arch protective shield made of plexiglass. The shield is placed between the undersurface of the vertical stainless steel tube and the bilateral posterolateral chest wall of the rat by adjustment of the height of the platform (Fig. 1). The specially designed arch on the undersurface of the shield averts direct contact between the rat spine, scapulae and the aluminum weight and directs the high-fall energy to the bilateral posterolateral chest wall of the rat. The friction throughout the study was not recorded.

### Method for reproducing the model

The rats were anesthetized with pentobarbital (40 mg/kg) injected peritoneally. The third thoracic vertebra and the eighth vertebra were marked superficially. The anesthetized rats were put on the platform in a prone position. The height of the platform was adjusted so as to place the protective shield in between the undersurface of the vertical stainless steel tube and the bilateral posterolateral chest wall of the rat. The spin was adjusted to allow the aluminum cylindrical weight (0.1 kg ×3) to fall freely onto the protective shield. Various heights and masses were adopted to generate 4 groups of injury energy of 2.1, 2.4, 2.7 and 3.0 J. Each group contained 9 rats. The injury energy of any group with the mortality of rats exceeding 20% was defined as lethal energy. Additional rats (n=9) were treated as the control group.

### Measurement of the contusion

**Figure 2:**
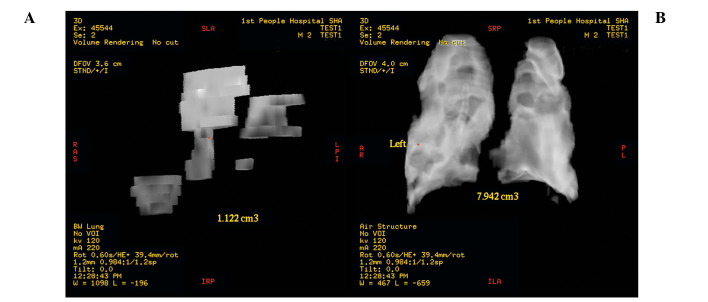
(A) Three-dimensional reconstruction of contusion in a rat showing the contusion volume to be 1.122 cm^3^. (B) Three-dimensional reconstruction of bilateral lungs showing total pulmonary volumes to be 7.942 cm^3^, demonstrating the contusion volume percentage to be 14.13%.

After the rats regained consciousness naturally, free activity in the cage was permitted and water was offered. After injury (4 h), the rats were anesthetized again with the method documented above and underwent high resolution computed tomography (CT) examination. The pulmonary opacity area, regarded as the PC lesion, on each slice of spiral CT scan was documented and three-dimensionally reconstructed by Advantage Workstation AW 4.3 computer software (GE Healthcare, Waukesha, WI, USA), so that the precise contusion volume could be measured (Fig. 2A). The bilateral pulmonary fields were then also reconstructed and the pulmonary volume was also measured (Fig. 2B). Total contusion volume for both pulmonary fields was expressed as a percentage of total pulmonary volume. Special attention was paid to the possibility of bone fracture and thoracic and peritoneal bleeding.

### Blood gas analysis

After CT examination, all of the living rats were administered 100% oxygen for 5 min. Then, a mid-line ventral incision was made to expose the descending aorta, from which 0.5 ml arterial blood was drawn into a heparinized syringe, followed by analysis with an ABL5 blood gas analyzer (Radiometer America, Westlake, OH, USA). The arterial oxygen partial pressure (PaO_2_) was documented. During dissection, special attention was paid to the possibility of bone fracture and thoracic and peritoneal bleeding.

### Histological assessment

After blood gas analysis, the rats from the group receiving maximal sublethral energy were sacrificed by transection of the abdominal inferior vena cava. The bilateral lungs and the heart were removed. The lung tissue where the opacity area was the most evident in the CT scan was sliced and immersed in 1% formalin together with the cardiac tissue. The tissue was then stained with hematoxylin and eosin (H&E) and viewed using optical microscopy. Histological assessment of cardiac tissue included evaluation of all four chambers of the heart in multiple sections. Special attention was paid to the findings at the cardiac apex, which was most likely to suffer from contrecoup injury. For those rats dying from the injury, autopsy was performed to determine the cause of death. The same experienced pathologist was blinded to the histological assessment.

### Statistical analysis

Statistical Product and Service Solutions (SPSS) 13.0 software was used to perform all statistical calculations. Unless otherwise stated, mean values and standard deviations are reported. For the comparison between means, a one-way analysis of variance (ANOVA) was used. For further intergroup pairwise comparisons, ANOVA with Bonferroni’s post hoc test was used. In the case of categorical variables, a Chi-square or a Fisher’s exact test was used when appropriate. Scatter-dot figure was drawn based on PaO_2_ and contusion volume percentage. Pearson’s correlation was used to analyze the relationship between the two parameters and the linear regression equation was fitted. The regression coefficient was checked by the Student’s t-test. For all statistical analyses, P-values of <0.05 were considered to indicate statistical significance.

## Results

### Mortality and determination of the maximal sublethal injury energy

The mortality in the 3.0 J group was as high as 33.3% (3/9), which exceeded the criterion of lethal energy (Table I). Autopsy showed that the cause of death was rib fracture and thoracic bleeding, which was also the cause of death for the only case of mortality in group 2.4 J. Thus, 2.7 J was regarded as the maximal sublethal energy in this model.

### Comparison of PaO_2_ and contusion volume percentage between groups

The PaO_2_ and contusion volume percentage were different among all groups and achieved significance (Table II). Upon further intergroup pairwise comparisons, the PaO_2_ and contusion volume percentage was not significantly different between groups 2.1 and 2.4 J. The difference in PaO_2_ between groups 2.7 and 3.0 J did not reach significance. Compared with those in the groups with lower energy, the rats in group 2.7 J manifested larger contusion lesion and more severe hypoxemia, which basically met the diagnostic criterion for acute respiratory distress syndrome (ARDS).

### Relationship between PaO_2_ and contusion volume percentage

**Figure 3:**
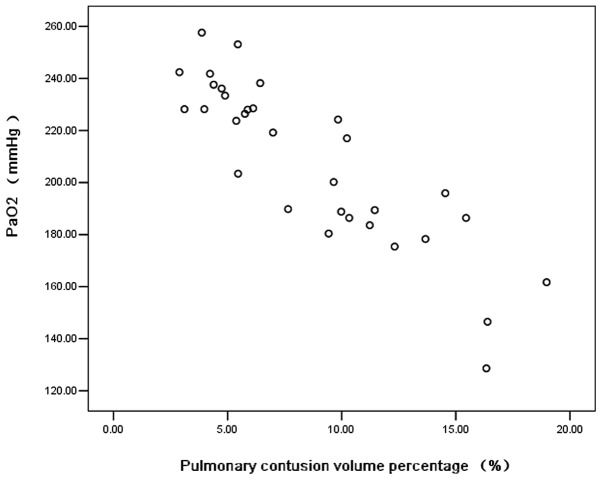
Relationship between PaO_2_ and contusion volume percentage 4 h after injury.

Pearson’s correlation showed that there was basically a negative correlation between PaO_2_ and contusion volume percentage (R^2^=0.762, Fig. 3). The linear regression equation was fitted, the efficacy of which was determined by the Student’s t-test (Table III): y =−6.18x + 261.57, where y represents PaO_2_ and x represents contusion volume percentage shown by three dimension (3D)CT.

### Histological assessment

**Figure 4:**
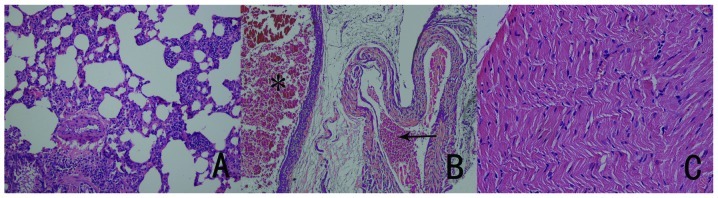
Histological assessment by H&E staining. (A) Infiltration of neutrophils; some monocytes and lymphocytes were apparent with pulmonary parenchyma and interstitial edema, and there was still red blood cell infiltration in the alveolar space; magnification, ×100. (B) Illustration of arterial (←) and venous congestion (*); H&E; magnification, ×40. (C) Representative section of cardiac apex showing no evidence of myocardiac disruption, edema and bleeding; magnification, ×200.

Pathological examination 4 h after injury showed numerous neutrophils together with several monocytes and lymphocytes apparent in the air space with pulmonary parenchyma and interstitial edema, and still red blood cell infiltration in the alveolar space; these observations were compatible with a diagnosis of PC (Fig. 4A). Arterial and venous congestion was noted in some areas (Fig. 4B). There was no significant evidence of cardiac muscle disruption, edema and bleeding in the multiple heart sections (Fig. 4C).

## Discussion

The earliest animal models for PC were reported in the 1960’s, when Border *et al* (3) induced PC in canines using a weight dropped on a shielded chest. The constancy of lungs was compromised, and the pathology examination revealed atelectasis, consolidation and air trapping, which confirmed the diagnosis of PC. Nichols *et al* (4) modified the model based on the work of Border by sliding steel bars with a plate onto the chest wall of canines. They observed the progression of PC in dogs and revealed that hypoxemia reached a peak at 24 h and was improved by 48 h. Other large animal models for PC such as swine and monkey have been developed (5). Although more similar to PC in humans, these large animal models are much more costly and lack molecular probes and other cell-and mediator-specific reagents, compared with rodent models. Thus, rodent models are widely used in the study of PC.

To date, several mature and reproducible rodent models for PC have been reported in the literature. Knoferl *et al* (6) developed the first murine model for PC with a unilateral chest impact using an ultrasonic blast wave. Hoth *et al* (7) published the results of another closed chest murine model, where an electrical cortical impactor was used to deliver a target energy level producing a uniform contusion to the right lung. These experimental platforms were found to be complex. Wang *et al* (8) induced combined cardiac and pulmonary contusion using a swing pendulum with energy ranging from 1.7 to 6.0 J. This model had advantages such as easy design and controllable energy, however, myocardial contusion inevitably had an impact on the interpretation of the results of hypoxemia. Moreover, the leading cause of murine mortality in their study was blunt myocardial contusion. Raghavendran *et al* (2) induced PC in anesthetized rats by dropping a weight onto a precordial protective shield to direct the impact force away from the heart and toward the lungs. Their experimental platform was suspended on Teflon guides, which was costly and relatively complex. However, compared with the animal models mentioned above, their models were easily reproducible, and injury energy was controllable. Moreover, the models were regarded as isolated PC models. Our model was created on the basis of Raghavendran’s work. The posterolateral chest wall of the anesthetized rat was wedged into the under surface of the arch shield by adjusting the height of the base on which the rat was fixed, so that the injury energy, which was dependent on the mass and height of the weight, could be transferred to the posterolateral chest wall of the rat. The rat was in a prone position, which increased the distance between the injury region and the heart and hence further reduced the risk of myocardial contusion. Contrecoup injury was once a concern in this model, but biopsy confirmed the diagnosis of bilateral PC and ruled out the existence of myocardial contusion. The model also used an impact induction method that was highly relevant to not only motor vehicle accident injury, but also to high fall injury, both of which are the most common causes of clinical blunt thoracic trauma. The value for the acceleration of gravity was taken to be 10 m/sec^2^ to facilitate calculation.

The energy in our model was higher than that in Raghavendran’s model. The elasticity of the posterolateral ribs of the rats is not as good as that of the anterolateral ribs. Therefore, a certain energy level induced a smaller contusion lesion size in our model when compared with the model of Raghavendran. Less elasticity is likely to facilitate rib fracture caused by high-fall energy, which was a major concern in our model. We prospectively chose 20% as the mortality limit to define sublethal PC injury energy. To direct the injury to the thorax and avoid neck and abdominal injury, we marked the third thoracic vertebra and the eighth thoracic vertebra prior to injury. The specially designed arch shield lowered the risk of vertebral and scapular fracture and mediastinal organ injury. Mortality in the 3.0 J group exceeded the limit, and the deaths of the rats were relevant to rib fracture and subsequent thoracic bleeding. which demonstrated that the energy was too high. However, energy <3.0 J is considered safe.

The major data collected in this study included PaO_2_ and PC volume percentage demonstrated by 3DCT. PaO_2_, which reflects the severity of hypoxemia, is the most manifest pathophysiological alteration in PC and is essential in experimental and clinical research on PC. Imaging is clinically the most common method used for the diagnosis of PC. Although expedient and common, chest plain reontography may overlook lesions shortly after injury. Research (9) which compared the results of plain chest radiography and pathologic results from autopsy found that plain chest radiography had a sensitivity of only 34%, which would be even lower if the film was taken shortly after injury (10). CT, when compared with plain film, was reported to have a high sensitivity of ∼100% (11). Its advantages rely on the strict correlation between CT density and the lung physical density, allowing a quantification of lung compartments with different degrees of aeration. Therefore, as far as early diagnosis is concerned, chest CT is the optimal tool. Estimation of contusion volume based on opacity area in bilateral lung fields on each slice is feasible. However, as for quantification, this method relies on visual estimation. Therefore, inter-reader and intra-reader variation may result in poor reproducibility in the quantification of PC volume. Attenuation-defined measurement has been reported (12). This method considered hydrothorax and atlectasis which were also likely to impair gas exchange. However, variation in the attenuation in different injury circumstances also made the definition of the damaged lung field difficult, and it was of no use in isolated PC models. PC volume percentage quantification demonstrated by 3DCT is accurate and repeatable, and thoracic bleeding, organ injury and bone fracture can be observed on the CT image simultaneously. A prospective research study (13) demonstrated that PC volume percentage quantification demonstrated by 3DCT was an independent predictor for ARDS development after PC. Thus, in this study, it was PaO_2_ and PC volume percentage demonstrated by 3DCT that were chosen as indictors for assessing the size of PC lesions and evaluating the severity of PC. Given the fact that there is probably pulmonary exudative lesions of various causes in ‘healthy’ rats which would mimick PC lesions in 3DCT imaging, we treated another 9 rats as controls to obtain data on the background of pulmonary exudation in ‘healthy’ rats.

A previous study (14) illustrated that clinical PaO_2_/FiO_2_ was not linearly correlated to PC volume percentage as demonstrated by 3DCT, which was explained by the fact that the PaO_2_/FiO_2_ ratio may be influenced by numerous factors such as the state of consciousness and systolic blood pressure. In this model, PaO_2_ and PC volume percentage were basically negatively correlated (Fig. 3, R^2^=0.762), which illustrated that 76.2% of the change in PaO_2_ was explained by a change in contusion volume percentage. That is, hypoxemia in this study reflected the severity and distribution of PC. Hence, compared with clinical PC which is accompanied by other injuries, PC induced by this model was regarded as simple and specific.

PC is an evolving lesion. Animal experiments and clinical research illustrate that hypoxemia in PC is serious 4 h after injury, may last for 24 h and improves by 48 h (2). We chose 4 h as the interval between injury and data collection, which minimized the impact of pulmonary infection, suptum retention and atlectasis on the results.

Cardiac output and other variables of cardiac dysfunction were not measured in the present study, since these are not specific to blunt cardiac trauma. The hypoxemia and pulmonary hemodynamic changes (specifically acute reactive pulmonary hypertension) associated with PC have been shown in many animal studies to seriously affect cardiac function (15). Multiple biopsies of cardiac muscles demonstrated the existence of spotted congestion change without apparent myocardial rupture, which is relevant to pulmonary hemodynamic changes during PC.

Finally, we focused on hypoxemia, distribution of lesions on imaging and the pathological findings of PC. Quantification of pulmonary inflammation and comparison with past models in the literature were lacking. However, this is a pilot study. After determining the maximal sublethal energy of this model, we will carry out a quantificative study on pulmonary inflammation after PC using this model in subsequent research.

In conclusion, the method documented in this study, which is easy to duplicate, results in satisfactory isolated bilateral pulmonary contusion in rats, and thus 2.7 J can be regarded as the maximal sublethal injury energy. The severity of hypoxemia reflected the distribution of PC lesions to a great extent.

## Figures and Tables

**Table I:** Mortality rate of each energy group (n=9).

Group	Number of deaths	Mortality (%)
2.1 J	0	0.0
2.4 J	1	11.1
2.7 J	0	0.0
3.0 J	3	33.3

**Table II:** Comparison in PaO_2_ and contusion volume percentage between groups.

	Control	2.1 J	2.4 J	2.7 J	3.0 J	P-value
PaO_2_ (mmHg)	397.22±20.99^a^	238.78±11.57	220.83±16.33^b^	193.93±16.68^d^	166.23±25.53^f^	<0.001
Contusion volume percentage	0.41±0.85^a^	4.46±1.08	5.88±1.16^c^	10.50±0.96^e^	15.90±1.84^g^	<0.001

a Compared with any other injury groups, P<0.001;

b compared with group 2.1 J, P=0.497;

c compared with group 2.1 J, P=0.172;

d compared with group 2.4 J, P=0.044;

e compared with group 2.4 J, P<0.001;

f compared with group 2.7 J, P=0.065;

g compared with group 2.7 J, P<0.001.

**Table III:** Linear regression coefficient^a^.

Variable	β	SE	Sβ	t-value	P-value
Constant	261.57	6.12	-	42.75	<0.001
Contusion volume percentage	−6.18	0.63	−0.87	−9.79	<0.001

β, regression coefficient; SE, standard error of regression coefficient; Sβ, standard regression coefficient.

a Student’s t-test.
